# Squat and Countermovement Vertical Jump Dynamics Using Knee Dominant or Hip Dominant Strategies

**DOI:** 10.5114/jhk/159285

**Published:** 2023-01-20

**Authors:** Keitaro Seki, Tomoki Nagano, Kiyohide Aoyama, Yasunori Morioka

**Affiliations:** 1 Department of Physical Education, College of Humanities and Sciences, Nihon University, Tokyo, Japan.; 2 Master’s Program of Education, Graduate School of Literature and Social Sciences, Nihon University, Tokyo, Japan.; 3 Department of Competitive Sports, College of Sports Sciences, Nihon University, Tokyo, Japan.

**Keywords:** strength training, joint torque, stretch-shortening cycle

## Abstract

This study aimed to investigate squat jump and countermovement jump kinetics in the knee dominant and hip dominant postures. Participants included 12 male sports science students. They were instructed to perform a squat jump and a countermovement jump with two squat postures: knee- and hip-dominant postures. The jumping motion and ground reaction force were recorded using a motion capture system and a force plate, respectively. A p-value of 0.05 was considered statistically significant. There was a significant interaction for the maximal knee joint extension torque, with the knee-countermovement jump being more than twice higher than that of other conditions, but not for mechanical work of the knee joint, which was significantly greater in the knee posture than in the hip posture. No significant interactions were found in mechanical work and maximal extension torque of the hip joint, both of which were significantly greater in the hip posture than in the knee posture, and in the countermovement jump than in the squat jump. This study showed that the effects of countermovement and posture were different for joints and that these effects were independent in the hip joint, but interacted in the knee joint. In the knee joint, the posture increased the effect of countermovement on extension torque, but the effect on mechanical work was small. This suggests that countermovement in the knee posture has little effect on the lifting work, but results in a great load on the knee extensors.

## Introduction

Squats are one of the most common exercises to develop strength and power of the lower limb muscles as well as an event of powerlifting; many athletes perform squats in their training routine (Chandler and Stone, 1991). Squats are practiced by a wide variety of people, ranging from athletes to the elderly, because they are simple and easy to perform. In particular, coaches consider squats as the best resistive exercise for athletes because they engage numerous muscles that are also involved in other athletic movements (McLaughlin and O’Shea, 1984).

Generally, the effect of resistance training is determined by the lifted load (intensity) and the number of repetitions. It is believed that a training protocol with high loads and a low number of repetitions is effective for improving muscle strength and one with medium loads and a high number of repetitions is effective when the aim is muscle hypertrophy (Choi et al., 1998). For squats, in addition to the lifted load and the number of repetitions, stance width (Escamilla et al., 2001), squatting depth (Bryanton et al., 2012), and the knee position (Fry et al., 2003) are variables to be considered depending on the training objective (Rippetoe, 2011). The load characteristics of the body during squats vary depending on these variables; especially, the posture at the bottom position would be an important variable. The effect of training would differ in different bottom position postures even when the lifted load and the number of repetitions are the same. Squat depth is a variable of the bottom position posture. A study by Bryanton et al. (2012) reported that the load on the knee joint extensors increased with increasing squat depth. The bottom position posture is defined not only by the squat depth, but also by the knee position. Fry et al. (2003) compared the lower limb joint kinetics during squats in different knee positions at the bottom position, and reported that greater knee joint torque and the lower hip joint torque were produced when in the knee dominant posture (i.e., the knee placed past the toe at the bottom position). Consequently, there is no doubt that the bottom position posture has a major effect on the load characteristics of the squat.

Meanwhile, countermovement also affects the load characteristics of squats. Squats consist of descending and ascending phases. It is believed that a stretch-shortening cycle (SSC) action occurs in the leg extensors at the bottom position, which is the moment when the direction of motion switches from descending to ascending (Rippetoe, 2011). However, there are only few studies on countermovement during squats. McBride et al. (2010a) reported a vertical impulse and jump height in static and countermovement jump squats using various squat depths and loads, but they did not directly compare static and countermovement jump squats. Manabe et al. (2007) examined joint kinetics between slow and quick squats, and indicated that the plantarflexion and extension torque of the knee and hip joints were significantly greater in quick squats than in slow squats. However, McBride et al. (2010b) reported little difference in kinetics and muscular activity between countermovement and isometric squats. These findings are inconsistent, but may be due to the bottom position posture. Manabe et al. (2007) controlled both the knee and the hip joint angle at the bottom position, while McBride et al. (2010b) only controlled the knee joint angle, and both studies examined squats in different postures. The bottom position posture needs to be controlled to examine the effect of countermovement during the squat.

The lifted weight should also be considered to examine the load characteristics of squats. However, it is difficult to determine a lifted weight that generates an equal load for all participants. Some studies (Bryanton et al., 2012; Escamilla et al., 2001; Lahti et al., 2018; Manabe et al., 2007) have used load setting based on the maximal lifted weight basis, and others (Cappozzo et al., 1985; McKean et al., 2010) based on body mass. It is therefore difficult to determine which approach is appropriate. Different lifted weights would have different effects on the bottom position posture, countermovement, and load characteristics. Therefore, we adopted a vertical jump as the experimental exercise. The vertical jump is considered a simpler version of squats; in particular, the take-off phase of the vertical jump and the ascending phase of squats have similar characteristics, because the aim of these exercises is to lift the body vertically. Some researchers have therefore used jumping to investigate squats (McBride et al., 2010a). In addition, it is easy to control experimental conditions, such as the bottom position posture and countermovement. This kind of the approach, which uses simpler and similar exercises, is called mimicking exercise (Schwameder, 2014), and it would be helpful for future studies and practical training. Therefore, the purpose of the present study was to determine the effects of countermovement and squat postures on lower limb joint kinetics during vertical jumping. We hypothesized that the effect of countermovement on kinetics would differ in the posture, i.e., that countermovement would enhance knee and hip joint kinetics in the knee-and hip-dominant postures, respectively.

## Methods

### Participants

The present study included 12 male students (age: 21.1 ± 0.9 years, body height: 1.72 ± 0.07 m, body mass: 72.8 ± 12.8 kg). They were recruited from the department of physical education at a university and provided written informed consent prior to participation in the present study. All participants were healthy and had no lower extremity injuries. This study was approved by the Ethics Committee of the College of Humanities and Sciences, Nihon University in accordance with the Declaration of Helsinki.

#### Procedures

**Figure 1:**
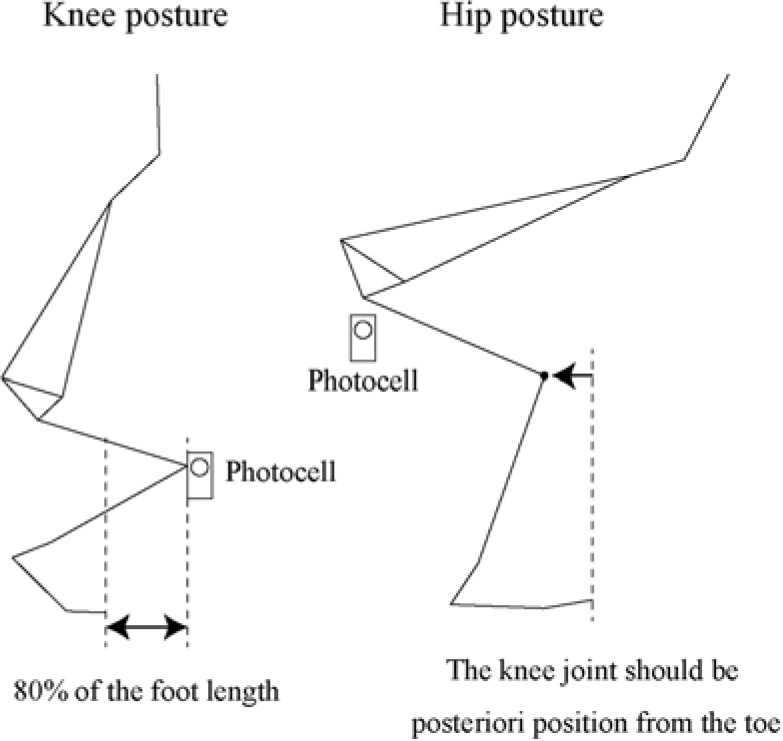
Summary of posture conditions at the lowest point of the body’s centre of mass.

Participants were asked to perform a vertical jump without an arm swing under four conditions. Jumping conditions were combinations of the posture at the lowest point of the body’s centre of mass (LCoM) and countermovement (Figure 1). The posture condition comprised knee and hip postures. The knee posture was defined as the knee joint located within 80% of the length of the foot anterior to the toe. The hip posture was defined as the hip joint moving posteriorly, and the shank maintaining a vertical posture. In both posture conditions, the thigh was parallel to the horizontal plane. Countermovement conditions comprised the countermovement jump (CMJ) and the squat jump (SJ) (Reiser et al., 2006). The CMJ was a vertical jump with countermovement from a standing posture. The SJ was a vertical jump from the squat posture, defined by the knee and hip postures. The vertical component of the ground reaction force (GRF) was monitored to avoid countermovement. Therefore, the jumping conditions were described as the Hip-CMJ, Hip-SJ, Knee-CMJ, and Knee-SJ. Participants were instructed to hold their arms at their hips to avoid the effects of the arm swing. The trial order was randomized on a subject-by-subject basis. Participants wore short tights and standard footwear (Wave Cruise 9, Mizuno, Japan) to avoid the effects of variation in footwear. Prior to the experiment, participants were familiarized with the exercises.

Kinematic data were recorded using a motion capture system with five cameras (Vicon Vero v2.2, Vicon Motion Systems, UK) at 250 Hz. Twelve reflective markers were attached to the right side of the body landmarks (the toe, fifth metatarsal bone, heel, lateral malleolus, lateral condyle, greater trochanter, anterior iliac spine, posterior iliac spine, hand, wrist, elbow, and shoulder). Marker placements were based on the estimation method of body segment inertial parameters (Ae et al., 1992). The markers were placed on the skin, except the following markers: the markers of the greater trochanter, anterior iliac spine, and posterior superior iliac spine were placed on the tights, and those of the toe, 5^th^ metatarsal bone, and heel were attached to the shoes. We assumed bilateral symmetry. GRF was measured using a force plate (9281B, Kistler, Switzerland) at a frequency of 1 kHz.

#### Analysis

Data were processed using MATLAB version 2020a (MathWorks Inc., Natick, MA, USA). We focused on the sagittal plane as it permits analysis of the effects of countermovement and the posture. The two-dimensional coordinates and GRF in the sagittal plane were smoothed using a Butterworth low-pass digital filter at 10 Hz (Bezodis et al., 2013). A rigid-body model consisting of 14 body segments (the head, torso, hand, forearm, upper arm, foot, shank, and thigh) was constructed using the smoothed coordinates of anatomical landmarks. The mass and the centre of mass location of each segment were estimated using the coefficients provided by Ae et al. (1992). Thereafter, the body’s centre of mass location (CoM) was obtained as the resultant centre of mass of all body segments. The ascending phase was defined as LCoM to take-off. The descending phase in the CMJ was defined as the instant at which the vertical velocity of the body’s CoM was negative for the LCoM. Jump height was defined as CoM height at the highest point. The joint torques of the ankle, knee, and hip were calculated using an inverse dynamics method (Winter, 1980). Mechanical work was calculated by integrating joint power, which is the inner product of the joint torque and joint angular velocity (Winter, 1983).

#### Statistical Analysis

Results are presented as the mean ± standard deviation (SD). Statistical analysis was performed using SPSS version 25.0 (IBM, Armonk, NY, USA). Normality of the variables was evaluated using the Kolmogorov-Smirnov test prior to any analysis. A two-way (posture × countermovement) repeated measures analysis of variance was used to test the main effects and interactions. Homogeneity of variances was evaluated using the Mauchly’s test of sphericity. The lack of sphericity was treated by adjusting the degrees of freedom before performing an F-test. When the interaction was significant, a simple main effect test and multiple comparisons were conducted. When the interaction was not significant, but the main effect was significant, multiple comparisons were conducted. Multiple comparisons were performed using the Bonferroni method. Variables during the descending phase were tested using a paired *t-*test because the descending phase was only in the CMJ. The level of statistical significance was set at 5%.

## Results

Table 1 demonstrates the spaciotemporal variables. There was no significant interaction and main effects in the jump. The ascending phase was significantly longer in the SJ than in the CMJ (partial η^2^ = 0.75, F = 33.71, *p* < 0.001, Table 1). The descending phase was significantly longer in the Hip-CMJ than in the Knee-CMJ (t = 2.25, *p* = 0.046).

**Table 1:** Mean (± SD) spaciotemporal variables in each condition.

Variables	Mean ± SD				Interaction F
	K-CMJ	K-SJ	H-CMJ	H-SJ	
Jump height (m)	1.42 ± 0.07	1.42 ± 0.08	1.43 ± 0.09	1.42 ± 0.08	0.48
Ascending phase duration (s)	0.37 ± 0.03	0.49 ± 0.12	0.36 ± 0.03	0.51 ± 0.11	0.29
Descending phase duration (s)	0.53 ± 0.06	-	0.58 ± 0.07	-	-

**Figure 2:**
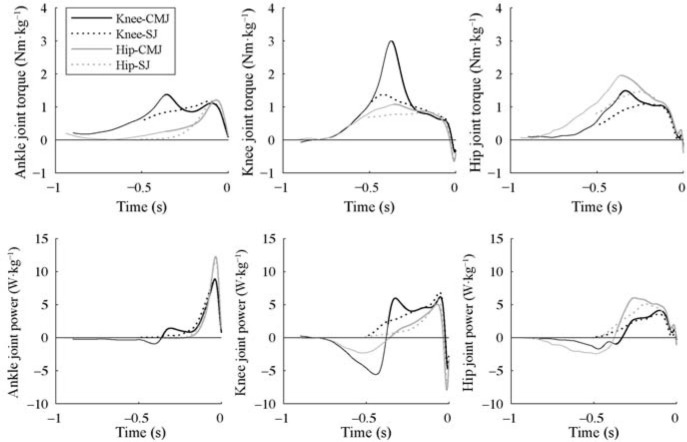
Mean instantaneous joint torques and joint power of the ankle, knee, and hip during descending (CMJ) and ascending phases of each jump. Data are aligned with respect to the moment of the take-off. The descending phase of the CMJ is illustrated with a thinner line.

Figure 2 shows joint torques and joint power of the ankle, knee, and hip joints during the descending and ascending phases under each condition. The ankle plantarflexor muscles showed maximal plantarflexion torque in the latter part of the ascending phase in most of the conditions, but only at the beginning of the ascending phase in the Knee-CMJ. A significant interaction was observed in the maximal plantarflexion torque (partial η^2^ = 0.47, F = 9.88, *p* = 0.009, Table 2), which was significantly greater in the Hip-CMJ than in the Hip-SJ (*p* = 0.005), in the Knee-CMJ than in the HipCMJ (*p* = 0.021), and in the Knee-SJ than in the HipSJ (*p* = 0.021) (Table 2). The knee extensor muscles exerted torque throughout the ascending phase under all conditions, with the maximal extension torque appearing early in the ascending phase. A significant interaction was observed in the maximal knee extension torque (partial η^2^ = 0.80, F = 42.54, *p* < 0.001, Table 2), which was significantly greater in the Knee-CMJ than in the Knee-SJ (*p* < 0.001) and Hip-CMJ (*p* < 0.001), and in the Knee-SJ than in the Hip-SJ (*p* < 0.001) (Table 2). The hip extensor muscles exerted torque throughout the ascending phase. The maximal hip extension torque appeared at the beginning of the ascending phase in the Knee-CMJ and the Hip-CMJ, and in the middle of the ascending phase in the Knee-SJ and the Hip-SJ. No significant interaction was found in the maximal hip extension torque (partial η2 = 0.05, F = 0.53, *p* = 0.482), but the main effects of the posture (partial η^2^ = 0.89, F = 86.51, *p* < 0.001) and countermovement (partial η^2^ = 0.59, F = 16.11, *p* = 0.002) were significant (Table 2). The maximal hip extension torque was significantly greater in the hip posture than in the knee posture (*p* < 0.001) and in the CMJ than in the SJ (*p* = 0.002) (Table 2).

**Table 2:** Mean (± SD) maximal joint torques and joint power of the ankle, knee, and hip under each condition.

Variables	Mean ± SD				Interaction F
	K-CMJ	K-SJ	H-CMJ	H-SJ	
Plantarflexion torque (Nm•kg^−1^)	1.43 ± 0.25	1.26 ± 0.10	1.22 ± 0.15	1.32 ± 0.09	9.88**
Knee extension torque (Nm•kg^−1^)	2.99 ± 0.65	1.45 ± 0.23	1.17 ± 0.31	1.02 ± 0.19	42.54***
Hip extension torque (Nm•kg^−1^)	1.66 ± 0.29	1.34 ± 0.22	2.01 ± 0.21	1.62 ± 0.16	0.53
Ankle joint power (W•kg^−1^)	9.01 ± 1.81	9.80 ± 2.32	12.43 ± 1.78	12.13 ± 1.84	9.88**
Knee joint power (W•kg^−1^)	7.84 ± 1.70	7.29 ± 2.37	5.63 ± 1.92	6.47 ± 2.39	1.96
Hip joint power (W•kg^−1^)	4.90 ± 1.91	4.71 ± 1.23	7.50 ± 1.29	6.52 ± 0.97	1.37

** : p < 0.01, ***: p < 0.001

Ankle joint power showed a similar pattern under all conditions (Figure 2), but a significant interaction was found in its maximal value (partial η^2^ = 0.47, F = 9.88, *p* = 0.009). Maximal ankle joint power was significantly greater in the Hip-CMJ than in the Knee-CMJ (*p* = 0.027), in the Hip-SJ than in the Knee-SJ (*p* = 0.021), and in the Hip-CMJ than in the Hip-SJ (*p* = 0.028). Knee joint power showed the maximal value before the take-off under all conditions except the Knee-CMJ, where it showed a bimodal pattern with a peak in the first half of the ascending phase. No significant interaction (partial η^2^ = 0.15, F = 1.96, *p* = 0.190) or main effect of countermovement (partial η^2^ = 0.01, F = 0.14, *p* = 0.714) was found in maximal knee joint power, but the main effect of the posture was significant (partial η^2^ = 0.52, F = 11.66, *p* = 0.006) (Table 2). Maximal knee joint power was significantly greater in the knee posture than in the hip posture (*p* = 0.006). Hip joint power showed a peaked trend under all conditions; however, the peak appeared earlier in the Hip-CMJ and later in the Knee-CMJ and the Knee-SJ (Figure 2). No significant interaction (partial η^2^ = 0.11, F = 1.37, *p* = 0.267) or main effect of countermovement (partial η2 = 0.15, F = 1.92, *p* = 0.193) was found, but the main effect of the posture was significant (partial η^2^ = 0.76, F = 34.94, *p* < 0.001) (Table 2). Maximal hip joint power was significantly greater in the hip posture than in the knee posture (*p* < 0.001).

**Figure 3:**
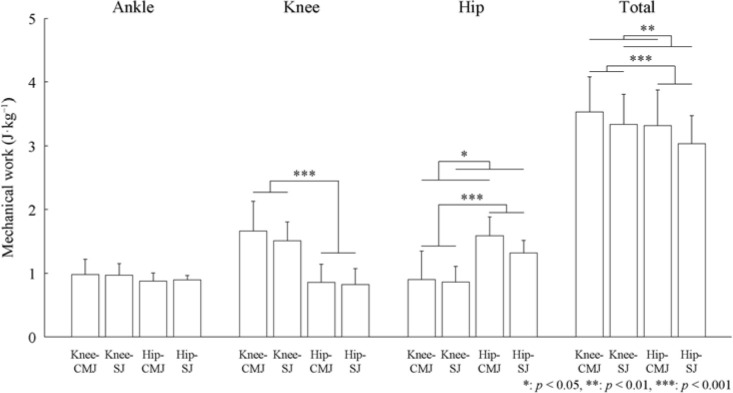
Mean (± SD) mechanical work of the lower limb joints during the ascending phase under each condition. *: p < 0.05, **: p < 0.01, ***: p < 0.001

Figure 3 demonstrates the mechanical work of the ankle, knee, and hip joints as well as the total mechanical work values of the lower limb joints. No significant interaction (partial η^2^ = 0.02, F = 0.19, *p* = 0.672) or main effects of posture (partial η2 = 0.19, F = 2.63, *p* = 0.133) and countermovement (partial η^2^ = 0.01, F = 0.10, *p* = 0.754) were found in the mechanical work of the ankle joint. No significant interaction (partial η^2^ = 0.06, F = 0.68, *p* = 0.427) or main effect of countermovement (partial η2 = 0.21, F = 2.97, *p* = 0.113) was found in the mechanical work of the knee joint, but the main effect of the posture was significant (partial η^2^ = 0.90, F = 99.58, *p* < 0.001). The mechanical work of the knee joint was significantly greater in the knee posture than in the hip posture (*p* < 0.001). No significant interaction (partial η^2^ = 0.16, F = 2.05, *p* = 0.180) was found in the mechanical work of the hip joint, but the main effects of the posture (partial η^2^ = 0.91, F = 115.85, *p* < 0.001) and countermovement (partial η^2^ = 0.36, F = 6.22, *p* = 0.030) were significant. The mechanical work of the hip joint was significantly greater in the hip posture than in the knee posture (*p* < 0.001) and the CMJ than in the SJ (*p* = 0.030). No significant interaction (partial η^2^ = 0.04, F = 0.51, *p* = 0.491) was found in the total mechanical work of the lower limb joints, but the main effects of the posture (partial η^2^ = 0.69, F = 24.33, *p* < 0.001) and countermovement (partial η^2^ = 0.65, F = 20.23, *p* = 0.001) were significant. The total mechanical work of the lower limb joints was significantly greater in the knee posture than in the hip posture (*p* < 0.001) and in the CMJ than in the SJ (*p* = 0.001).

## Discussion

The major findings of the present study were as follows: 1) there was a significant interaction for the maximal knee joint extension torque; 2) mechanical work of the knee joint was significantly greater in the knee posture than in the hip posture; 3) there was no significant interaction for the maximal hip joint extension torque; 4) mechanical work of the hip joint was significantly greater in the hip posture than in the knee posture and in the CMJ than in the SJ. These results supported the hypothesis for the knee joint, but not for the hip joint.

The interaction between the posture and countermovement was most clearly observed for maximal knee extension torque. In the Knee-CMJ, the knee extension torque was surprisingly greater than under the other conditions, with its value being approximately twice as high, while that of the Knee-SJ was also significantly greater than that of the Hip-SJ. In addition, the mechanical work of the knee joint was significantly greater in the knee posture than in the hip posture, but its value was not twice as high. Fry et al. (2003) reported the effect of the posture on barbell squats, with the knee extension torque being greater and the hip extension torque being less in the knee dominant posture than in the hip dominant posture. In the knee posture, the knee joint is located within 80% of the length of the foot anterior to the toe. This posture would increase the moment arm of the ground reaction force to the knee joint, which would also relate to greater knee joint torque and mechanical work. In the present study, negative knee joint power was twice as high during the descending phase in the Knee-CMJ than in the HipCMJ. Evidently, the Hip-SJ and the Knee-SJ did not show this kind of negative joint power because these conditions started from a static squat posture. It is well known that the SSC action caused by countermovement enhances force during the concentric phase following the eccentric phase, and this is related to the magnitude of negative power (Bosco et al., 1981; Komi and Nicol, 2011). The maximal knee extension torque in the Knee-CMJ would be due to the SSC action, namely countermovement. The present study suggests that the knee joint torque is the most affected variable by countermovement as well as the posture in vertical jumping.

As shown above, there were some significant differences in kinetics between the Knee-CMJ and the Hip-CMJ, but none in jump height. Jump height has been reported to be greater in the CMJ than in the SJ (Asmussen and Bonde-Petersen, 1974; Kozinc et al., 2021), although the present study did not show a significant difference in this regard. However, the difference in the maximal knee joint extension torque was excessively large. One explanation for this might be the extreme posture at the LCoM. The present study adopted extreme postures as compared to regular squat exercises to differentiate between posture conditions, which may have allowed for greater knee extension torque, but not an effective vertical acceleration of the body. The differences in joint power and mechanical work of the knee joint between the Knee-CMJ and Knee-SJ were smaller than those of the maximal knee extension torque. These results also suggest that the effectiveness of the knee joint torque in the Knee-CMJ might be lower as compared to the other conditions.

Interestingly, no significant interactions were found between the maximal extension torque and mechanical work of the hip joint. The kinetics of the hip joint would have a significant interaction similar to that of the knee joint, but the results were not consistent with our hypothesis. The maximal hip extension torque was significantly greater in the CMJ than in the SJ. Manabe et al. (2007) reported that the hip extension torque was significantly greater in squats with countermovement than in squats without countermovement. They suggested that a greater hip extension torque was caused by the SSC action of the gluteus maximus muscle (Manabe et al., 2007). In addition, the maximal hip extension torque was significantly greater in the hip than in the knee posture. This result was similar to that of a previous study (Fry et al., 2003). The independent effects of countermovement and the posture might be due to the gluteus maximus muscle being a monoarticular muscle. The knee extensor muscles include mono- and bi-articular muscles: the rectus femoris muscles. Manabe et al. (2007) reported that muscle activities of the rectus femoris, as well as the vastus lateralis, were significantly greater in squats with countermovement. The effect of the posture is greater on the force production of biarticular muscles compared to monoarticular muscles. Therefore, the effects of countermovement and the posture were independent in the hip joint, where monoarticular muscles acted predominantly, but may have interacted in the knee joint, where the biarticular muscles were involved.

The present study showed that the effects of countermovement and the posture were different for joints; they worked independently in the hip joint, but interacted in the knee joint during vertical jumping. In the knee dominant posture, the effect of countermovement would be enhanced on the knee joint. Under this condition, the knee joint extension torque was dramatically greater, but its mechanical work did not change. This implies that an excessively great knee joint torque does not contribute to getting up from the squatting position; it only increases the load on the knee joint. However, in the hip joint, the effect of countermovement was not affected by the posture. This phenomenon can be applied during squats and should be examined in future studies.

## References

[ref1] Ae, M., Tang, H.-p., & Yokoi, T. (1992). Estimation of inertia properties of the body segments in japanese athletes. Society of Biomechanisms Japan, 11, 23–33.

[ref2] Asmussen, E., & Bonde-Petersen, F. (1974). Storage of elastic energy in skeletal muscles in man. Acta Physiologica Scandinavica, 91(3), 385–392. 10.1111/j.1748-1716.1974.tb05693.x 4846332

[ref3] Bezodis, N. E., Salo, A. I. T., & Trewartha, G. (2013). Excessive fluctuations in knee joint moments during early stance in sprinting are caused by digital filtering procedures. Gait Posture, 38(4), 653–657. 10.1016/j.gaitpost.2013.02.015 23540768

[ref4] Bosco, C., Komi, P. V., & Ito, A. (1981). Prestretch potentiation of human skeletal muscle during ballistic movement. Acta Physiologica Scandinavica, 111(2), 135–140. 10.1111/j.1748-1716.1981.tb06716.x 7282389

[ref5] Bryanton, M. A., Kennedy, M. D., Carey, J. P., & Chiu, L. Z. F. (2012). Effect of Squat Depth and Barbell Load on Relative Muscular Effort in Squatting. Journal of Strength and Conditioning Research, 26(10), 2820–2828. 22797000 10.1519/JSC.0b013e31826791a7

[ref6] Cappozzo, A., Felici, F., Figura, F., & Gazzani, F. (1985). Lumbar spine loading during half-squat exercises. Medicine and Science in Sports and Exercise, 17(5), 613–620. 4068969

[ref7] Chandler, T. J., & Stone, M. H. (1991). The Squat Exercise in Athletic Conditioning: A Position Statement and Review of the Literature. Strength and Conditioning Journal, 13(5), 51–58.

[ref8] Choi, J., Takahashi, H., Itai, Y., & Takamatsu, K. (1998). The difference between effects of “power-up type” and “bulk up type” strength training exercises—with special reference to muscle cross-sectional area, muscular strength, anaerobic endurance. Japanese Journal of Physical Fitness and Sport Medicine, 47, 119–130.

[ref9] Escamilla, R. F., Fleisig, G. S., Lowry, T. M., Barrentine, S. W., & Andrews, J. R. A. (2001). A three-dimensional biomechanical analysis of the squat during varying stance widths. Medicine & Science in Sports & Exercise, 33(6), 984–998. 11404665 10.1097/00005768-200106000-00019

[ref10] Fry, A. C., Smith, J. C., & Schilling, B. K. (2003). Effect of knee position on hip and knee torques during the barbell squat. Journal of Strength and Conditioning Research, 17(4), 629–633. 14636100 10.1519/1533-4287(2003)017<0629:eokpoh>2.0.co;2

[ref11] Komi, P. V., & Nicol, C. (2011). Stretch-Shortening Cycle of Muscle Function. In Neuromuscular Aspects of Sport Performance (pp. 15–31). Wiley-Blackwell. 10.1002/9781444324822.ch2

[ref12] Kozinc, Z., Zitnik, J., Smajla, D., & Sarabon, N. (2021). The difference between squat jump and countermovement jump in 770 male and female participants from different sports. European Journal of Sport Science, 22(7), 1–9. 10.1080/17461391.2021.1936654 34075858

[ref13] Lahti, J., Hegyi, A., Vigotsky, A. D., & Ahtiainen, J. P. (2018). Effects of barbell back squat stance width on sagittal and frontal hip and knee kinetics. Scandinavian Journal of Medicine and Science in Sports. 29(1), 44–54. 10.1111/sms.13305 30230052

[ref14] Manabe, Y., Shimada, K., & Ogata, M. (2007). Effect of slow movement and stretch-shortening cycle on lower extremity muscle activity and joint moments during squat. Journal of Sports Medicine and Physical Fitness, 47(1), 1–12. https://www.ncbi.nlm.nih.gov/pubmed/17369791 17369791

[ref15] McBride, J. M., Kirby, T. J., Haines, T. L., & Skinner, J. (2010a). Relationship Between Relative Net Vertical Impulse and Jump Height in Jump Squats Performed to Various Squat Depths and With Various Loads. International Journal of Sports Physiology and Performance, 5(4), 484–496. 10.1123/ijspp.5.4.484 21266733

[ref16] McBride, J. M., Skinner, J. W., Schefer, P. C., Haines, T. L., & Kirby, T. J. (2010b) Comparison of kinetic variables and muscle activity during a squat vs. a box squat. Journal of Strength and Conditioning Research, 24(12), 3195–3199. 21132859 10.1519/jsc.0b013e3181f6399a

[ref17] McKean, M. R., Dunn, P. K., & Burkett, B. J. (2010). Quantifying the Movement and the Influence of Load in the Back Squat Exercise. Journal of Strength and Conditioning Research, 24(6), 1671–1679. 20508473 10.1519/JSC.0b013e3181d8eb4e

[ref18] McLaughlin, T. M., & O’shea, C. (1984). Coaches Roundtable: The squat and its application to athletic performance. Strength and Conditioning Journal, 6(3), 10–23.

[ref19] Reiser, R. F. I., Rocheford, E. C., & Armstrong, C. J. (2006). Building a Better Understanding of Basic Mechanical Principles Through Analysis of the Vertical Jump. Strength and Conditioning Journal, 28(4), 70–80.

[ref20] Rippetoe, M. (2011). *Starting strength* (3rd edition ed.). The Aasgaard Company, Wichita Falls, TX, USA.

[ref21] Schwameder, H. (2014, 12–16 July). Concepts in ski jumping biomechanics and potential transfer to other sports 32^nd^ International Conference of Biomechanics in Sports, Johnson City, TN, USA.

[ref22] Winter, D. A. (1980). Overall principle of lower limb support during stance phase of galt. Journal of Biomechanics, 13, 923–927. 7275999 10.1016/0021-9290(80)90162-1

[ref23] Winter, D. A. (1983). Moments of force and mechanical power in jogging. Journal of Biomechanics, 16(1), 91–97. 6833314 10.1016/0021-9290(83)90050-7

